# Public Use of Complementary Medicine for Children in Saudi Arabia: A Questionnaire-Based Cross-Sectional Study

**DOI:** 10.7759/cureus.46689

**Published:** 2023-10-08

**Authors:** Meshari Attar, Essam I Jastania, Rayan Mgarry, Hassan Alshaikh, Yaser M Alsinnari, Ziad M Bukhari, Mohammed S Alqarni, Sara S Abed

**Affiliations:** 1 Internal Medicine, Al-Mandiq General Hospital, Albaha, SAU; 2 Emergency Medicine, Al Makhwah General Hospital, Al Makhwah, SAU; 3 College of Medicine, King Saud Bin Abdulaziz University for Health Sciences, Jeddah, SAU; 4 Pediatrics and Child Health, Security Forces Hospital - Makkah, Mecca, SAU; 5 Family Medicine, Yanbu General Hospital, Ministry of Health, Yanbu, SAU; 6 Internal Medicine, Prince Mohammed Bin Abdulaziz National Guard Hospital, Madinah, SAU; 7 Ophthalmology, King Abdulaziz Medical City, Ministry of National Guard Health Affairs, Jeddah, SAU; 8 Internal Medicine, National Guard Hospital, King Abdulaziz Medical City, Jeddah, SAU; 9 Research and Development, King Abdullah International Medical Research Center, Jeddah, SAU; 10 Pediatrics, King Abdulaziz Medical City, Ministry of National Guard Health Affairs, Jeddah, SAU

**Keywords:** attitude, knowledge, children, saudi arabia, alternative medicine, complementary medicine

## Abstract

Background and objectives: The use of complementary and alternative medicine (CAM) is growing among adults and children. Extensive data is available regarding the pattern and frequency of CAM used in adults in Saudi Arabia, but limited data is available for children. This study aims to examine the level of knowledge, attitude, and practice about the use of CAM in the pediatric population in Saudi Arabia.

Subjects and methods: A cross-sectional descriptive study on the use of CAM in children was carried out in the general population of Saudi Arabia. Data was collected by non-probability consecutive sampling technique through an online-based questionnaire from 132 participants. In addition, data analysis was done using IBM’s Statistical Package for the Social Sciences (SPSS). The data collected consisted of socio-demographic details, knowledge, attitude, and practice of CAM in children.

Results: CAM was reported in all 132 participants (100%), with 45% (N=59) using it without informing their physicians. The mean age of the children was 17 months old, 55.3% (N=73) children were males, and 44.7% (N=59) were females. The most common form of CAM used was herbal medicine, 91% (N=120), while alternative medicine was used in 16.7% (N=12) of the children. Honey was the most used herb (68.2%, N=90), followed by anise (65.2%, N=86), Zamzam water (holy water) (59.1%, N=78), and olive oil (56.8%, N=75).

Conclusions: The use of CAM is very common for children in the general population of Saudi Arabia, with herbal medication being the most common. This constitutes a dire need to regulate this field and provide enough information for the public and health care practitioners to provide the best health care. In addition, future awareness campaigns are needed to bridge the communication gap between parents and physicians and provide better information about the benefits and safety of CAM use.

## Introduction

With the development of evidence-based science and the birth of modern medicine in the 20th and 21st centuries, complementary and alternative medicine (CAM) has been scrutinized. With more literature pouring in, authors defined complementary medicine as being outside the boundaries of contemporary medicine and focused on the negative connotations associated with this care. These extensive negative connotations with complementary medicine were mainly subjected to its unscientific philosophy [1]. With the rise of popularity and use of CAM, a consensus-based definition was devised by professionals from contemporary medicine and alternative medicine specialists, herbalists, acupuncturists, chemists, and clinical psychologists as "Complementary medicine is diagnosis, treatment and/or prevention which complements mainstream medicine by contributing to a common whole, by satisfying a demand not met by orthodoxy or by diversifying the conceptual frameworks of medicine” [2].

CAM includes a variety of therapies, herbal medications, alternative methods, and physiotherapeutic modalities and may constitute more than 150 different modalities [3]. Three major classes of complementary or alternative medicine (CAM) being used have been identified in a study in Denmark: 1) herbal medicine, (2) alternative therapy, and (3) chiropractic [4]. Herbal medicine includes four subgroups: herbal drugs, vitamin and mineral supplements, extracts, and various unclassified herbal preparations. Alternative therapy comprises unconventional remedies such as acupuncture, healing, cupping, cauterization, kinesiology, and reflexological therapy. In contrast, chiropractic consists of adjustment exercises for the spine to deal with alignment and back issues. Other CAM modalities identified through other studies are craniosacral, osteopathy, aromatherapy, massage, reiki, and Buteyko breathing [4]. Moreover, phytotherapy, manual-based therapies, energy medicine, traditional Chinese medicine, bioelectrical medicine, body-mind therapies, and anthroposophical medicine have also been identified as CAM [5].

CAM practice has been rising worldwide, and different authors have determined its prevalence. Surveys conducted globally to determine its prevalence have been analyzed by Ernt in a systematic review of the literature, with results ranging from 9% to 65% and maybe growing [6]. The prevalence of use of CAM shows wide diversity among different countries and in different surveys in the same countries, as shown by Ernst, due to discrepancies in defining CAM, different demographic characteristics and attitudes of the people, and different types of CAM; thus, care must be taken while generalizing these results [6].

With the rise of CAM use in the adult population, the prevalence and practices of CAM are also rising in children worldwide. A systematic review of the literature on the prevalence of CAM use in children showed variable results with a rise in popularity [7]. Extensive data is available regarding the prevalence of CAM in adults, especially those with chronic or terminal illnesses [8]. UK has 41.1%, Europe has 56%, Bahrain has 63%, Saudi Arabia has 75%, and Turkey has 41.1%, while similar extensive data is not available for the child age group [9-13]. Some national surveys were conducted to determine the use of CAM in children in developed countries, including Europe, which showed a 52% prevalence of CAM in Children, the UK with 17.9%, and the USA with 15% prevalence [8,13]. Because of the rise in CAM use, the American Academy of Pediatrics has dedicated a task force to determine the extent and pattern of CAM use in children [14].

With the lack of a national survey for the prevalence of CAM in Saudi Arabia, several studies in different regions have shown the prevalence of CAM use to be 75% in the general population [15-18]. A review of the surveys done in Saudi Arabia put the prevalence range between 21.6% and 90.5%, which was well above the worldwide prevalence of 9% to 65% [6,19]. Most studies conducted in Saudi Arabia showed the use of CAM for the treatment of liver disease, skin diseases, neurological disorders, and chronic illnesses like diabetes and asthma, with Quran recitation and honey being the most common form of CAM used in Saudi Arabia [12,20-21]. The out-of-pocket cost of CAM use in Saudi Arabia is estimated to be 8.2 billion USD [18].

With the rise in CAM use in adults, prevalence is also rising in children worldwide, significantly impacting child health care. Similarly, this rise in the popularity of CAM should be valid for Saudi Arabia. However, there is no data available for Saudi Arabia to assess the patterns and extent of CAM practices in children. We aim to determine the true prevalence, attitude, and practice of people regarding the use of CAM and the side effects of CAM use in the pediatric population in Saudi Arabia that can be generalized to the whole population. This research will help understand the implications of CAM use on child health care and make strategies for the education, research and development, and regulation of the CAM field.

## Materials and methods

This was a cross-sectional questionnaire-based survey for the use of CAM in children taken from December 2021 to February 2022 in the National Guard Health Affairs in Jeddah, Saudi Arabia. This study was approved by the Institutional Review Board (IRB) at King Abdullah International Medical Research Center (approval number: #IRBC/420/09).

Data was collected by structured online and interview-based questionnaires taken from parents/guardians/caretakers based on a non-probability consecutive sampling technique. There were no inclusion and exclusion criteria for study subjects, and consent was taken at the start of the questionnaire to participate in the study. Ethical consideration was taken. All information taken remained confidential and secure in a password-protected computer. The data was only accessible to the personnel conducting the study.

The questionnaire was designed based on three major portions: 1) socio-demographic details, including age, gender, nationality, and the number of children; 2) CAM use open-ended questions, including if they have used any form of CAM, type of CAM used, frequency of CAM used; 3) attitudes, such as "why they started using CAM?," "Are the children suffering from any disease?." The questionnaire was designed with extensive literature research and validated by medical education experts. A pilot study was done to check for the difficulties in administering the questionnaire, and the question's clarity was discussed with other researchers, and modifications were made according to the feedback from the pilot study. The reliability and internal consistency of the questionnaire were done using Cronbach's alpha. The sample size was calculated using a 0.05 probability of type I error, a 0.05 precision, and a prevalence proportion of 0.179, with a 15% estimated prevalence rate of CAM use in children and a 70% response rate.

Responses were typed into Microsoft Excel, and statistical analysis was done through SPSS statistics software. Qualitative variables like gender and education were presented as frequency, percentage, and bar graphs, while quantitative variables were presented as mean and standard deviation. Independent t-test and chi-square test were used to compare the data. The dependent variable is alternative medicine use, and the independent variable is the knowledge and attitude of people. A p-value of <0.05 was taken as significant. After the statistical analysis, a result was generated that can be generalized to a larger population.

## Results

One hundred thirty-two online and interview responses were received during the study period (58.4% of the sample size). The mean age of the responding caregiver was 36 years (SD=7), and the mean age of the children was 17 months. The caregivers' education level was high school (91%, N=120) and bachelor's degree (9%, N=12). 55.3% (N=73) of children were males, and 44.7% (N=59) were females. 97% (N=129) of the responders had Saudi nationality. All the responders used complementary medicine for their children at least once. In the survey, complementary medicine was divided into alternative and herbal medicine. Alternative medicine was used in 22 children with a rate of 16.7%. Alternative medicine includes the use of cupping in 7.6% of the children (N=10), cauterization in 5.3% (N=7), acupuncture in 3% (N=4), massaging in 0.8% (N=1), and casting in 0.8% (N=1). Of the 22 alternative medicine users, 91% were using it one to three times per year. Herbal medicine was used in 120 children's responders at 91%. Different types of herbs were used, as shown in Table 1. 

**Table 1:** Types of herbal medicine

Types of herbal medicine	N (%)
Honey	90 (68.2%)
Anise	86 (65.2%)
Zamzam water	78 (59.1%)
Olive oil	75 (56.8%)
Sesame oil	47 (35.6%)
Black seeds	45 (34.1%)
Ginger	41 (31.1%)
Cloves of garlic	31 (23.5%)
Caraway	28 (21.2%)

Herbal medicines were used three to six times per year by 33% of the users, 14% were used monthly, 23.3% were used weekly, 7.5% were used daily, and 20% were used only when needed. Seventeen children had chronic diseases, and eight of the chronic disease children were using complementary medicine as the primary treatment of their disease. In addition, 45 (34%) responders agreed on using complementary medicine without informing the physician, as shown in Table 2. 20% gave reasons for use because of improvement with the previous use of CAM in children.

**Table 2:** Reasons to use complementary medicine without informing doctors

Reasons to use complementary medicine without informing doctors	N (%)
Good outcome and improvement with the previous usage	9 (20%)
It is natural and has no side effects	8 (17.7%)
It is not essential to tell the doctor	6 (13.3%)
Doctors do not believe in complementary medicine	2 (4.4%)
The treatment prescribed by the doctor did not improve the symptoms	2 (4.4%)
Emergency use and no time to inform doctors	1 (2.2%)
No reasons	17 (37.7%)

36 (27.3%) agreed that the use of complementary medicine is better than the treatment the doctors prescribed, as shown in Table 3. 

**Table 3:** Reasons why complementary medicine is better than prescribed medications

Reasons why complementary medicine is better than prescribed medications	N (%)
It is natural and has no side effects, unlike medications	15 (41.6%)
Complementary medicine cures the disease; medications treat the symptoms only	4 (11.1%)
Complementary medicine can treat any disease; medications are specific for one disease	1 (2.7%)
Complementary medicine can improve the immunity, unlike medications	1 (2.7%)
No reasons	21 (41.6%)

Other responses about the previous experience of the caregiver of using complementary medicine and the expected outcomes are shown in Table 4.

**Table 4:** Experience of the caregiver of using complementary medicine

Question	Answer	n (%)
Did complementary medicine treat your child's symptoms?	Yes	73 (55.3%)
	No	32 (24.2%)
	I do not know	27 (20.5%)
Do you use complementary medicine as a primary or alternative use?	Primary use	73 (55.3%)
	Alternative use	59 (44.7%)
What is your expected outcome of using complementary medicine?	Improvement	99 (75%)
	Worsening	2 (1.5%)
	No change	31 (23.5%)
Did your child develop side effects after using complementary medicine?	Yes	24 (18.1%)
	No	108 (81.9%)

Allergic reactions were seen in eight (6.1%) children after using complementary medicine, diarrhea in six (4.5%), vomiting in three (2.3%), asthmatic reaction in three (2.3%), nausea in two (1.5%), and headache in two (1.5%).

## Discussion

Our result showed that all the participants in our study used CAM for their children at least once (100%), with 45% using it without informing their physician. These results are consistent with another Saudi survey in pediatric outpatient clinics showing that 90% of the 131 patient’s parents reported using CAM in their children [22]. This is well above the national average for the use of CAM in the general population of Saudi Arabia, which is 70%, taken with the previous data available [15-18]. However, most of these studies included participants above 16 years. A family survey in the Riyadh region by Gad et al. showed that 37.3% of families reported using CAM for their children, with Quran recitation being the most common type used [23]. Another survey in the outpatient department in Jeddah reported that 42% of the families reported using CAM for their children, with religious and spiritual healing being used in 82% of the cases. A study similar to ours was done in Palestine, with a mean child age of three to four years, and found that all participants also used some kind of CAM at least once for their children. A similar study in Turkey showed an 83% prevalence of CAM use in the pediatric outpatient department [24]. Compared to national surveys among other countries, Simpson and Roman's study in the UK showed that 17.9% of children under 16 used CAM at least once [8]. The national average of children's use of CAM in the USA was found to be at 8% to 15%, Canada at 11%, and Denmark at 53% [4,24-25]. Data from 18 countries in Europe showed a 52% prevalence of CAM use among children [10]. Most of the studies conducted worldwide on children's use of CAM are not representative of the general population and include samples limited to a health care unit (pediatric ward, outpatient clinic) or in children with chronic illnesses; thus, these results cannot be generalized. Madsen et al. showed that children with the following conditions are more likely to be using CAM: gastrointestinal diseases among infants (50%), joint diseases (36%), premature infants (25%), and children suffering from central nervous system diseases (24%), among others [4]. A study in Washington DC, USA, showed that 21% of parents used CAM for their children, which may not be representative of the population [26]. According to the American Academy of Pediatrics, the use of CAM was reported to be high among children from low socioeconomic status or with children suffering from a chronic disease and with special needs. Children with asthma, attention deficit hyperactivity disorder (ADHD), autism, and juvenile arthritis reported the most use of CAM [14]. In Children with neurological disorders, CAM was used in 42% of participants visiting pediatric neurology clinics, while a survey conducted on patients with juvenile arthritis shows that about 70% of the patients used unconventional remedies or therapies to control their disease [27-29]. 

In the dominant Muslim society of Saudi Arabia, the religion and Arabic literature have a major role in influencing the type of CAM used, with Quran recitation being the most common, as shown by Al-Faris et al. [15]. In our study, herbal medicine was more commonly used than alternative medicine, with a prevalence of 91% (N=120). Among the herbal medicine, honey (68.2%, N=90), anise (65.2%, N=86), and zamzam water (holy water) (59.1%, N=78) were among the most commonly used, with most participants using herbal medicine every week. These findings were consistent with other regional studies, showing that herbal medicine was the most common form of CAM in Saudi Arabia [16-18]. In the multistage household survey done by Al-Faris et al. in 2008 in the Riyadh region of Saudi Arabia, honey was the most commonly used herbal medicine and has also been used to treat liver disease [15,20]. The most common reason for using CAM for children was connected to good previous records and improvement of their ailments. However, in the household survey of Al-Faris, perceived failure of medical treatment and lack of trust in the Saudi health system was found to be an actual cause of CAM use [15]. Reasons for using or denying CAM as identified by Vlieger et al. were patients wanted to feel better, they preferred "natural" therapy, patients were hoping for a cure, some patients chose to use fewer medications compared to the conventional medical treatments, people were influenced by friends and family, good past experiences with unconventional therapies, and disappointment in the traditional treatments [5]. Similarly, in our study, 27.3% believed CAM to be better than prescribed medication and believed "it is natural and has no side effects.'' 55.3% replaced CAM as their primary treatment. In Denmark, 7% of users of herbal remedies replaced their medical treatment with herbal medicine, 12% replaced it with alternative therapy, and 25% replaced it with chiropractic [4].

55.3% of the patients saw improvement in their child's symptoms with CAM use, while 75% expected a positive outcome from CAM use. However, 18.1% of children developed side effects due to CAM use. The most common side effect encountered was allergic reaction, followed by diarrhea and vomiting. A study in Denmark highlighted 56% positive effects in qualitative analysis for herbal medicine, 79% with the use of alternative medicine, and 92% with chiropractic [4]. However, side effects were reported to be associated with herbal medicine in only 2% of users, 7% of users of alternative medicine, and 17% of chiropractic users, as reported by the patients, and no clinical confirmation was done in that regard. In addition, a comprehensive study identified the risks and adverse effects of acupuncture and Chinese herbal medicine. The study reports that adverse events related to acupuncture are fainting during treatment, increased pain, nausea and vomiting, localized skin infections, psychiatric disturbances, convulsions, and respiratory adverse events like pneumothorax [5]. Moreover, adverse effects related to Chinese herbal medicine were reported to be altered gastrointestinal habits leading to severe and continuous diarrhea, vomiting, and abdominal pain; fainting and dizziness; significant skin reactions; palpitations; severe fatigue; high blood pressure; psychiatric disturbances; central nervous system effects such as numbness or palsy; referral to a medical facility; jaundice; hepatotoxicity; renal toxicity; significant respiratory disturbances; and death [5].

The high public use of CAM in children and non-disclosure of this information heed the need for more general population education about the potential risks and benefits associated with this practice in children. In developing countries like Saudi Arabia, there is a need for policy-making, regulation, research, and development in the CAM field to cater to consumers' high demand and provide the best health care to the public. Physicians should use this topic to care best for their patients, and due to the high prevalence of CAM use, they should be asked about their patient history so that unnecessary side effects and drug reactions can be avoided. CAM should be incorporated into the health care system, and health care should be made accessible to reduce the need for alternatives.

There were limitations to our study as well. The study was done at the end of the COVID-19 pandemic, with most questionnaires being admitted online, while the remaining was done with face-to-face interviews to get more accurate results. Various biasing and chances of error exist with the online methodology. With a small sample size and predominant online study setting, care must be given to generalize this study to the general population. The problems encountered by similar surveys were identified and rectified within our study, including a consensus definition, and the option of open-ended questions to include all the possible types of CAM.

## Conclusions

The increased public use of CAM for children in Saudi Arabia poses a challenge for health care providers and the general public. Honey, anise, and cupping were among the most commonly used CAM modalities. This poorly regulated market can result in a public health (unwanted effect) in children due to adverse effects, as mentioned in the study. Developed countries, like Europe, have regulated this field with accessibility and integration with the health care system, proper policy-making, and education with research and development on the benefits and side effects of CAM.

It is necessary to cater to the increased demand for CAM and provide guidelines for CAM use to both public and health care practitioners to make the best decisions regarding health care. This alarms the health care providers to be aware of the high prevalence of CAM use in children and must be considered when they provide health care for the patient. Finally, appropriate awareness of the matter is needed to reduce the communication gap between physicians and parents about their children’s health care, increase accessibility to the health care system, and provide alternative evidence-based medical treatments to avoid unregulated remedies that can cost a child’s life.

## References

[1] Ernst E (1993). Scrutinising the alternatives. Lancet.

[2] Ernst E, Resch KL, Mills S (1995). Complementary medicine—a definition. Br J Gen Pract.

[3] (1996). Complementary medicine is booming worldwide. BMJ.

[4] Madsen H, Andersen S, Nielsen RG, Dolmer BS, Høst A, Damkier A (2003). Use of complementary/alternative medicine among paediatric patients. Eur J Pediatr.

[5] Vlieger AM, van de Putte EM, Hoeksma H (2006). The use of complementary and alternative medicine in children at a general paediatric clinic and parental reasons for use. Europe PMC.

[6] Ernst E (2000). Prevalence of use of complementary/alternative medicine: a systematic review. Bull World Health Organ.

[7] Ernst E (1999). Prevalence of complementary/alternative medicine for children: a systematic review. Eur J Pediatr.

[8] Simpson N, Roman K (2001). Complementary medicine use in children: extent and reasons. A population-based study. Br J Gen Pract.

[9] Posadzki P, Watson LK, Alotaibi A, Ernst E (2013). Prevalence of use of complementary and alternative medicine (CAM) by patients/consumers in the UK: systematic review of surveys. Clin Med (Lond).

[10] Zuzak TJ, Boňková J, Careddu D (2013). Use of complementary and alternative medicine by children in Europe: published data and expert perspectives. Complement Ther Med.

[11] Khalaf AJ, Whitford DL (2010). The use of complementary and alternative medicine by patients with diabetes mellitus in Bahrain: a cross-sectional study. BMC Complement Altern Med.

[12] Aboushanab T, Khalil M, Al Ahmari Y (2019). The present state of complementary medicine regulation in Saudi Arabia. J Integr Med.

[13] Gözüm S, Tezel A, Koc M (2003). Complementary alternative treatments used by patients with cancer in eastern Turkey. Cancer Nurs.

[14] Kemper KJ, Vohra S, Walls R (2008). The use of complementary and alternative medicine in pediatrics. Pediatrics.

[15] Al-Faris EA, Al-Rowais N, Mohamed AG (2008). Prevalence and pattern of alternative medicine use: the results of a household survey. Ann Saudi Med.

[16] Elolemy AT, Albedah AM (2012). Public knowledge, attitude and practice of complementary and alternative medicine in Riyadh region, Saudi Arabia. Oman Med J.

[17] Kazmi S, Sami W, Alharbi S (2018). Prevalence and perception of Cam usage in Majmaah, Kingdom of Saudi Arabia. Majmaah J Heal Sci.

[18] AlBedah AM, Khalil MK, Elolemy AT (2013). The use of and out-of-pocket spending on complementary and alternative medicine in Qassim province, Saudi Arabia. Ann Saudi Med.

[19] Alrowais NA, Alyousefi NA (2017). The prevalence extent of Complementary and Alternative Medicine (CAM) use among Saudis. Saudi Pharm J.

[20] Al-Zahim AA, Al-Malki NY, Al-Abdulkarim FM, Al-Sofayan SA, Abunab HA, Abdo AA (2013). Use of alternative medicine by Saudi liver disease patients attending a tertiary care center: prevalence and attitudes. Saudi J Gastroenterol.

[21] Mohammad Y, Al-Ahmari A, Al-Dashash F, Al-Hussain F, Al-Masnour F, Masoud A, Jradi H (2015). Pattern of traditional medicine use by adult Saudi patients with neurological disorders. BMC Complement Altern Med.

[22] Albassam AA, Ain MR, Alanazi SN, Alruthia Y, Alkharfy KM (2012). Use of complementary and alternative medicine in pediatric patients in Saudi Arabia. J Herb Med.

[23] Gad A, Al-Faris E, Al-Rowais N, Al-Rukban M (2013). Use of complementary and alternative medicine for children: a parents' perspective. Complement Ther Med.

[24] Araz N, Bulbul S (2011). Use of complementary and alternative medicine in a pediatric population in southern Turkey. Clin Invest Med.

[25] Davis MP, Darden PM (2003). Use of complementary and alternative medicine by children in the United States. Arch Pediatr Adolesc Med.

[26] Spigelblatt L, Laîné-Ammara G, Barry Pless I (1994). The use of alternative medicine by children. Am Acad Pediatr.

[27] Ottolini MC, Hamburger EK, Loprieato JO (2001). Complementary and alternative medicine use among children in the Washington, DC area. Ambul Pediatr.

[28] Al-Rumayyan A, Alqarni H, Almanna BS, Althonayan N, Alhalafi M, Alomary N (2020). Utilization of complementary medicine by pediatric neurology patients and their families in Saudi Arabia. Cureus.

[29] Southwood TR, Malleson PN, Roberts-Thomson PJ (1990). Unconventional remedies used for patients with juvenile arthritis. Am Acad Pediatr.

